# Advances in Random Fiber Lasers and Their Sensing Application

**DOI:** 10.3390/s20216122

**Published:** 2020-10-28

**Authors:** Hong Chen, Shaohua Gao, Mingjiang Zhang, Jianzhong Zhang, Lijun Qiao, Tao Wang, Fei Gao, Xinxin Hu, Shichuan Li, Yicheng Zhu

**Affiliations:** 1 Key Laboratory of Advanced Transducers and Intelligent Control System, Ministry of Education, Taiyuan University of Technology, Taiyuan 030024, China; chenhong0834@link.tyut.edu.cn (H.C.); gaoshaohua@tyut.edu.cn (S.G.); zhangjianzhong@tyut.edu.cn (J.Z.); qiaolijun@tyut.edu.cn (L.Q.); wangtao@tyut.edu.cn (T.W.); gaofeiphd@163.com (F.G.); huxinxin0909@link.tyut.edu.cn (X.H.); lishichuan0765@link.tyut.edu.cn (S.L.); zhuyicheng0866@link.tyut.edu.cn (Y.Z.); 2 College of Physics and Optoelectronics, Taiyuan University of Technology, Taiyuan 030024, China

**Keywords:** random fiber laser, fiber, random laser, optical fiber sensing

## Abstract

Compared with conventional laser, random laser (RL) has no resonant cavity, reducing the requirement of cavity design. In recent years, the random fiber laser (RFL), a novel kind of RL, has made great progress in theories and experiments. The RFL has a simpler structure, a more flexible design, and higher reliability. It has valuable applications for earth sciences, biological life sciences, and national defense security, due to these unique properties. This paper reviews the development of RFLs in the last decade, including their configurations based on various optical fibers and their output properties, especially the method of control. Moreover, we also introduce their applications in the optical fiber sensing system, which is a very important and practical orientation to study. Finally, this paper presents the prospects of RFLs.

## 1. Introduction

‘Laser’ is an abbreviation of ‘light amplification by stimulation emission of radiation’ [1]. This originates from an atom or molecule that can produce stimulated emission of photons [2,3]. In the 1950s, N. G. Basov et al. realized microwave amplification by stimulation emission of radiation (MASER), based on the theory by Einstein [4,5,6]. In 1960, stimulated optical radiation in ruby was achieved by Maiman et al. [7]—this indicated that the era of the laser had arrived. A conventional laser scheme [8] comprises three key elements: A pump, an active medium, and a resonator. The pump is used to provide power for the population inversion of the active medium, and the active medium is utilized to offer optical amplification to compensate for losses. There are two functions for the resonator: The first is the mode selection, which means only a single or a few longitudinal modes can oscillate to improve the coherence of lasers; and the second is that the resonator offers the feedback of the longitudinal mode. Lasing can be generated when total gain overcomes total loss in the cavity. Therefore, the spectral and temporal performances of conventional lasers are determined by the active medium and the resonator. On the contrary, random lasers (RLs) [9,10,11] have no well-defined mirror configuration like the Fabry-Perot (F-P) cavity [12], where feedback depends on multiple scattering. The concept of self-generation of light in a random scattering medium without cavity was introduced by Letokhov in 1968 [13,14]. It ignited the interest in the fascinating field of random lasing [15,16]. In 1994, Lawandy et al. introduced laser action in strongly scattering media for the first time [17]. In 1995, the discussion of Lawandy’s experiment [17] showed that random lasing has developed into an exciting area of research [18,19]. Different types of materials, such as powders, liquid crystals, quantum dots, biological tissues, and polymers, have been used to realize random lasing [20,21,22,23,24]. The spectral and temporal characteristics of RLs are defined by the randomly embedded localized spatial modes that can coexist with non-localized extended modes [25]. Usually, RLs have simple structures and complex emission properties. Therefore, RLs have gotten considerable attention in the last two decades. They have potential applications in speckle-free imaging, holography, and biology. Especially in the sensing area, we can tune the interplay of random media and cavity to optimize sensing applications [26,27]. Distributed feedback (DFB) lasers were first represented in 1971, which were referred to as mirrorless by authors [28]. Random DFB lasers utilize random Bragg gratings as feedback medium [29], while RLs utilize various disorder medium, including random Bragg gratings for feedback. However, the directionality and efficiency of emission are challenges in the development of RLs’ applications. Since optical fiber can transversely confine light in the fiber core, it is extensively used to improve RLs’ performance.

De Matos et al. [30] filled rhodamine 6G solution doped with TiO_2_ nano-particles in the hollow photonic crystal fiber (PCF) core and realized the first RFL in 2007, in which the realization of random lasing combined optical fiber waveguide and random distributed nano-particles. This opens the door of a novel kind of RL. In 2010, an alternative approach that utilized the intrinsic disorder to obtain random feedback was proposed by Turitsyn et al. [31], in which Rayleigh scattering and stimulated Raman scattering (SRS) provide feedback and gain, respectively. After that, RFLs have gotten much more development in optical science and technology [32,33,34,35,36,37,38,39], such as the physical mechanism of the RFLs [40,41], optical communication and optical sensing [42,43,44,45,46], speckle-free imaging [47], and high power [48,49,50,51,52,53].

However, the pump power is high up to watt-level for the operation of the all-fiber RFLs [31], due to the low Rayleigh backscattering coefficient of the SMF, leading to the narrow application. Experimental attempts on the threshold-decreasing approaches [54] have been performed continually. Using the fiber Bragg gratings (FBGs) with high reflection is an effective method. For example, a half-open RFL structure [55] was proposed, which FBG is on the one side of the RFL scheme. In this way, the central wavelength of the output beam is determined by the FBG, and the FBG provides much stronger feedback than that of optical fiber. Thus, the threshold of a half-open RFL scheme is nearly two times lower than that observed in a completely open cavity [39]. Another approach that improved gain by active fiber was considered, such as erbium [56] or ytterbium-doped [57,58] fiber, which could serve to decrease the threshold of the RFLs and shorten the length of optical fiber. As a result of the existence of rare-earth ion, the power of the pump source drops to the order of milliwatt magnitude. Moreover, RFLs of higher efficiency and the lower threshold would be obtained with strong enhanced random feedback of random fiber grating (RFG) from Rayleigh scattering, by increasing the index changes along with the fiber to the order of 10^−4^~10^−5^. The intensity of random scattering is three orders of magnitude more than that of the Rayleigh scattering. Nevertheless, the fabrication of RFG requires complex technical conditions and processes. It may take a lot of time, but cannot work out. Moreover, the femtosecond laser with ultra-short pulse width and very strong instantaneous power can induce nonlinear multi-photon absorption of materials to modulate the refractive index of the fiber [59,60]. An alternative approach of introducing strong backscattering, a segment of random reflectors artificially controlled, was inscribed in the fiber by using a femtosecond laser micromachining system. This process is less complicated than that of the fabrication of RFG, which can be realized by two methods. In detail, the first is that the pulse energies are from 0.32 to 0.36 mJ and the number of adjacent reflectors on the same position is less than 10. The second is that the pulse energies range from 0.32 to 0.4 mJ and the number of adjacent reflectors on the same position is less than 2 [61,62]. As the number of random reflectors increases, the random feedback is enhanced, but together with a larger loss in the system. Therefore, an appropriate number of reflectors that should be optimized to obtain a better output beam of the RFLs.

Even though the high power emission of the RFLs is restricted by the low pump source power [63,64], many studies have been carried out to improve its experimentally [65]. Take the way of gain as an example. If only considered SRS and stimulated Brillouin scattering (SBS) in the fiber, it requires a long distance to compensate for the loss. Thus, advances in the change of gain medium have been carried out to improve the efficiency of the pump, such as the active gain. Moreover, the application of shorter fibers is preferable for high-efficiency generation, in which the power of the output beam is in the level of hundreds of watts. Optical conversion efficiency can be high up to 94%, which is close to the quantum limit (95%) [49]. There is no doubt that the power of the output emission cannot ever exceed the power of the pump in all approaches. The most direct and effective way is that optimizing the configuration of the power pump source. Later on, various studies on the pump scheme, including both symmetric and asymmetric configuration, are used to find the optimal structure. Increasing the power of one-side pumped RFLs or using a two-side pump source is considered, making the output power reach hundreds of watts [39]. However, temporal instability of the output lasing would happen, due to the self-pulsing property of the coherent pump source. Passive fiber could be utilized to stabilize the pump source, but with the imperfect signal-to-noise ratio (SNR), which is less than 30 dB [66]. In 2017, Pu Zhou et al. used a broadband incoherent amplified spontaneous emission (ASE) source as the pump and implemented a stable output that more than 100W. However, there still exists some incomplete pump wave conversion even at the maximum pump power, leading to the low SNR less than 30 dB [67]. After that, researchers have found that the line-width of the ASE source should be responsible for the uncovered pump waves. In 2019, Pu Zhou et al. optimized the bandwidth (~10 nm) of the ASE source and obtained high power output with high SNR of 39 dB, which the spectral of purity is 99.96% [68]. The optimized scheme with bandwidth-adjustable ASE pumping provides an effective way to obtain high power and high SNR RFLs. Moreover, there are other practical approaches for optimizing the output beam [69,70] of RFLs. It will be covered in more detail in the later section. With optimized output 3 mission characteristics, RFLs have gotten wide applications in telecommunication, remote sensing, and distributed sensing.

This review concentrates on the advances in studies of RFLs and their applications in sensing. Until now, varieties of optical fibers have been used in the systems of the RFLs, which could provide optimal schemes for their applications. Just like conventional lasers, emission power, wavelength, and line-width are of great importance for the RFLs. Moreover, the optimization of RFLs is of practical importance for their widespread applications in industry. Sensing is one of the most important potential applications for RFLs, such as remote sensing and distributed optical fiber sensing. In the end, we summarize the progress of RFLs in the last decade and make the prospect for future research.

## 2. RFLs Based on Various Optical Fibers

It is quite remarkable that RFLs based on kinds of optical fibers would have distinct output properties, owing to corresponding features for different optical fibers. A variety of optical fibers, such as PCF, single-mode fiber (SMF), active optical fiber (AOF), dispersion compensation fiber (DCF), polarization-maintaining fiber (PMF), multimode fiber (MMF), tapered optical fiber (TOF), and true wave fiber (TWF), can be applied to rather different families of RFLs, which could be helpful for better understanding the mechanism of random lasing in the disordered system and even greatly broaden the application of RFLs. Therefore, investigations on the RFLs based on different kinds of optical fibers are expected to be very necessary.

### 2.1. PCF-Based RFLs

Light performs nonlinear effects, and pattern properties influenced by the arrangement of pores in the cladding, and birefringence effect—caused by the asymmetric arrangement of pores, while it transmits in the PCF [71,72,73,74]. This concentrates it in a set of tiny pores arranged periodically along the fiber. Thus, an unknown world that RFLs are based on the PCF has opened up.

**Figure 1:**
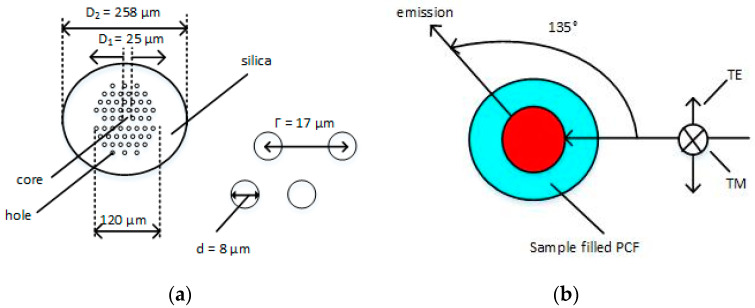
(**a**) Cross-section of the photonic crystal fiber (PCF); (**b**) optical setup for probing laser emission. TE, transverse electric; TM, transverse magnetic.

In 2007, a quasi-one-dimensional RFL generated by putting suspension TiO_2_ rhodamine 6G solution into the hollow-core PCF [30], and the efficiency was at least two orders of magnitude higher than similar systems in bulk format. This work has made researchers concentrate on the RFLs. Moreover, PCF is a promising platform of quasi-two-dimensional the RFLs, which could be convenient for understanding the oscillation mechanism of the RFLs. In 2015, random laser action was observed by filling 4-(dimethylene)-2-methyl-6-(4-dimethyl-styrene)-4h-pyran laser dye solution in PCF [75]. In 2017, Nagai et al. found that the threshold of the RFLs pump is related to the excitation polarization when filling PCF holes with dye-doped nematic liquid crystals (NLC). If so, the threshold would be much lower, due to stronger scattering efficiency caused by the high refractive index of the NLC. The cross-section of the PCF is shown in Figure 1a, and the optical setup for probing laser emission is depicted in Figure 1b [76].

### 2.2. SMF-Based RFLs

SMF is an alternative approach to form the RFL, avoiding the complex operation of filling the fiber with the material. RFLs based on SMF could be applied in long-distance and high-capacity fiber communication systems, fiber local area networks, and fiber sensors. The gain of SMF-based RFLs has two obvious principles: SRS and SBS.

**Figure 2:**
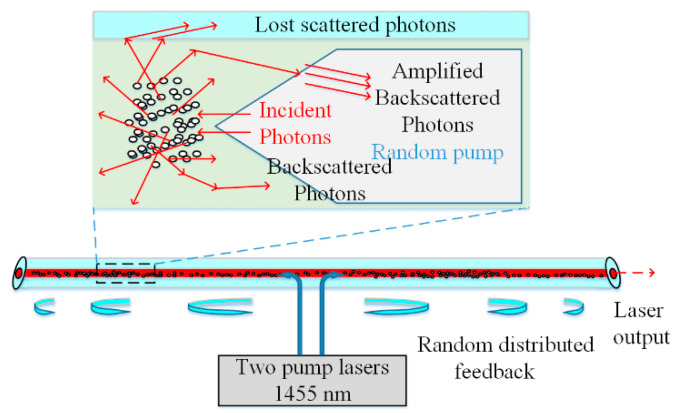
Principle scheme of random distributed feedback fiber laser operation.

A random distributed feedback laser based on common SMF with a length of 83 km was proposed in 2010 [31], of which SRS provides the gain. The operation principle scheme of random distributed feedback fiber laser is illustrated in Figure 2. Two lasers with a wavelength of 1455 nm act as bi-directional pumping sources at the center of SMF. Random Rayleigh scattering provides random feedback, due to irregularity of the SMF, and backward Rayleigh feedback light is stimulated by Raman amplification continuously. Eventually, lasing emits from the two sides of the SMF. One of the biggest advantages of this setup is that integration of feedback and gain would limit light in one direction, compared with the previous RLs. Moreover, the threshold of this structure is lower than that of structures at other wavelengths because the minimum loss window of SMF is at the wavelength of 1550 nm (0.22 dB/km). Moreover, the proposal of this innovation has inspired the application of the RFLs in communication, as the traditional communication window is about at the wavelength of 1550 nm.

**Figure 3:**
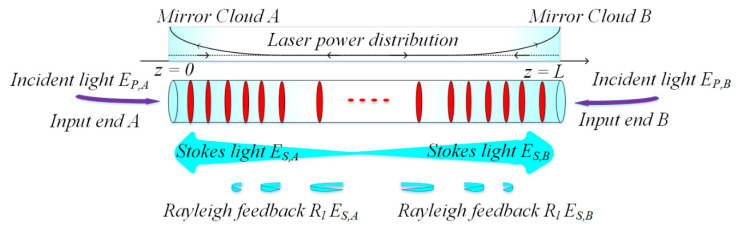
Configuration of random fiber F-P resonator-based Brillouin random fiber laser (RFL).

Even though the threshold of the RFLs [31] is at a low-level compared with the same setup of which output is not at the wavelength of 1550 nm, the minimum pump power is still at the order of watts. If the gain mechanism of the RFLs is SBS, the threshold of the pump will decrease to the order of milliwatts. Because the Brillouin gain coefficient is much higher, it is possible to provide enough amplification in the case of extremely week random feedback. As shown in Figure 3, two beams at two ends are pumps. Scattering stokes light travels along the opposite direction and agglomerates on both ends of the fiber, because the length of fiber is relatively short and instant pump power is higher. Moreover, because of random distributed Rayleigh scattering caused by inhomogeneity of SMF, the cavity mirror is formed along the optical fiber. However, valid Brillouin stokes feedback only occurs in the pump’s deleted regions, i.e., “mirror cloud” at the ends of SMF [77]. This, therefore, suggests that SMF can make up not only Raman RFLs but also Brillouin RFLs.

SMF-based RFLs that take advantage of Brillouin gain can obtain much narrower line-width emitted radiation than that of the Raman gain, which is due to the narrower bandwidth of the Brillouin gain. The research group of Bao has made significant investigations on SMF-based Brillouin RFLs. In 2013, they firstly observed a narrow-line-width peak of coherent Brillouin RFL, in which the line-width is ~10 Hz [78]. Theoretical and experimental research of coherent Brillouin RFL for frequency stability was reported in Reference [79], and the line-width of RFL is 50 Hz, but still relatively narrow. To understand the lasing mechanism of this kind of laser, they studied the frequency and intensity of noise characteristics of the Brillouin RFL based on bidirectional pumping [80]. In 2016, they presented a tunable ultranarrow-line-width microwave Brillouin RFL (under 3 dB bandwidth, the line-width is less than 10 Hz), which could provide a simple and economical design for microwave source [81]. This expands the application of the Brillouin RFLs. In Reference [82], six order stokes random lasing was realized with a high optical SNR of ~47 dB.

### 2.3. AOF-Based RFLs

Unlike SBS or SRS in optical fibers, rare-earth ions are real luminescent centers and can provide a greater gain for optical fibers. Therefore, the RFLs based on AOF can effectively reduce the threshold of the laser and shorten the length of the fiber. It constitutes an indispensable part of the field of the RFLs.

**Figure 4:**
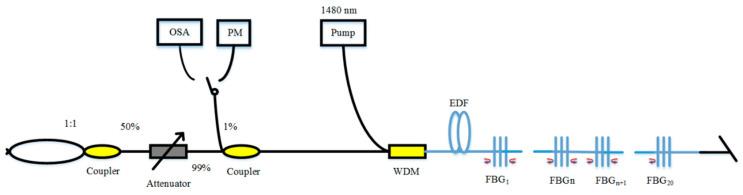
Schematic diagram of the experimental setup of the RFL based on erbium-doped fiber (EDF). OSA, optical spectrum analyzer; PM, power meter.

The erbium-doped RFL is the most common AOF-based RFL [83,84,85,86] because erbium ion has a corresponding energy level around at the wavelength of 1550 nm and a wide gain spectrum. In 2009, a low-threshold (3 mW) erbium-doped RFL was prepared by writing random grating in the erbium-doped fiber (EDF) [84]. In 2016, stable mode output of the erbium-doped RFL was performed by heating FBGs at specific locations in EDF and changing the location of the hot spots, and schematic diagram of the experimental setup of the RFL based on EDF is shown in Figure 4 [85]. In 2018, lasing with the line-width less than 10 kHz was obtained by adding EDF into the Brillouin RFL [86]. The length of EDF used in the RFL is shorter than that of SMF.

**Figure 5:**
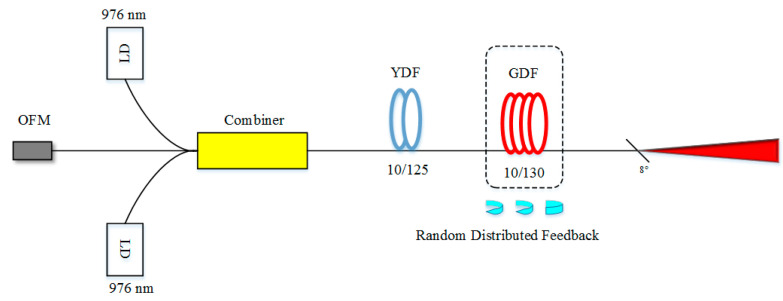
Experimental setup of the super-continuous (SC) source. LD, laser diode; OFM, optical fiber mirror.

Research of the erbium-doped RFL focuses on the C-band, while additional element-doped RFLs further extend the range of the optical band. For example, as the gain spectrum of ytterbium ion is relatively wide at the wavelength of 1060 nm, super-continuous (SC) spectrum from the visible to the near-infrared area was realized [58]. Figure 5 shows the experimental setup of the SC source. 1 km germanium-doped fiber (GDF) and 5 m ytterbium-doped fiber (YDF) are the hybrid gain medium, and 1 km GDF is the random feedback medium. Two 976 nm diodes are pump source with the power of 21.8 W and 22.9 W, respectively. The optical reflector provides all-optical feedback with the central wavelength of 1060 nm and the bandwidth of 40 nm, which could enhance the output of the SC spectrum. Moreover, the laser at the wavelength of 2000 nm was generated in 2016, in which an RFL at the center wavelength of 1173 nm was used to pump Tm-doped fiber [87]. There was no ASE emission or parasitic oscillation in the experimental results, due to the 1173 nm random distributed feedback fiber laser.

### 2.4. DCF-Based RFLs

**Figure 6:**
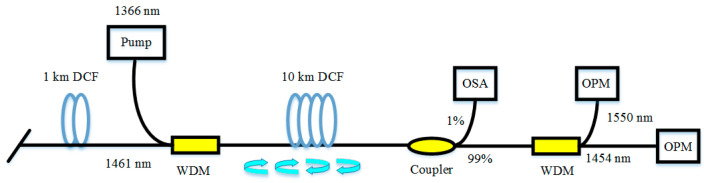
The schematic setup of the RFL is based on dispersion compensation fiber (DCF). OPM, optical power meter.

**Figure 7:**
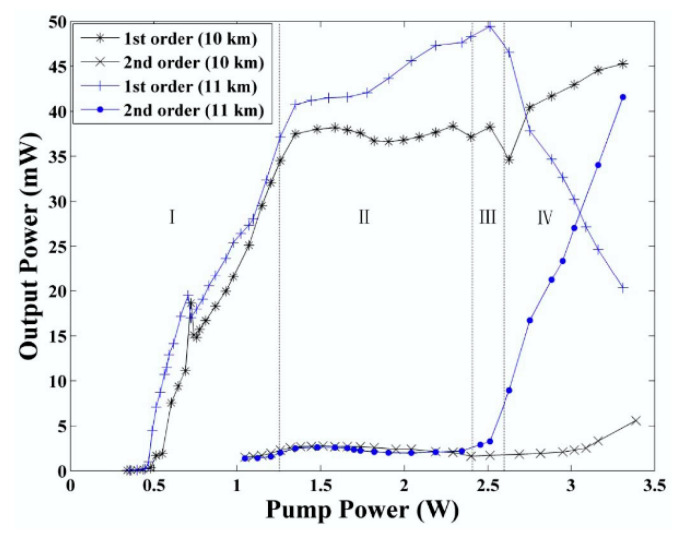
Output power as a function of pump power [88].

Because of its large negative dispersion, 1 km dispersion compensation fiber (DCF) can compensate for the dispersion of 4~8 km standard SMF. The RFL based on the DCF was first investigated in 2014 [88]. The experimental results demonstrated that the threshold of first-order output emission is only 0.45 W. The schematic setup of the RFL based on DCF is depicted in Figure 6. The pump light with the wavelength of 1366 nm is launched into 10 km DCF via 1365/1461 nm wavelength division multiplexer (WDM). The 1 km DCF provides additional Rayleigh scattering feedback for the RFL. The route is also revealed with the increase of pump power from first-order stokes light to second-order stokes light, which experiences three chaotic regimes, as shown in Figure 7.

Moreover, the position or length ratio of DCF and SMF has a great influence on the output of the RFLs (i.e., laser threshold, spectral stability, and shape). Lasing intensity can be considerably enhanced when the DCF locates at a higher pump power distribution position along the fiber [89]. These results are useful to understand the role of DCF in the RFLs and optical amplification. These studies provide theoretical supports for the flexible design and the optimization of the RFLs.

### 2.5. PMF-Based RFLs

**Figure 8:**
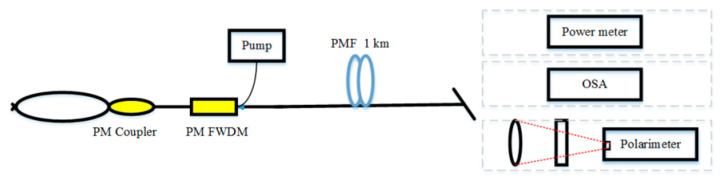
The scheme of all polarization-maintaining fiber (PMF) Raman RFL.

Standard SMF would have random birefringence, due to external mechanical stress in the practical application [90,91]. Thus, the polarization of light varies irregularly as it travels through fibers. However, for PMF, the strong birefringence and the short beat result in a high-quality polarization state of the transmitted light. A linearly polarized Raman RFL based on 1 km PMF was demonstrated in 2015 [92]. The result showed the pump quantum efficiency of third-order Stokes light exceeds 80%. The scheme of all-PMF Raman RFL is shown in Figure 8. The pump is linearly polarized with a wavelength of 1050 nm, which propagates in the PMF through a 1050/1100 nm PM filtered WDM (FWDM). A PMF loop mirror formed by a fused PMF is spliced with a coupler (1050 nm) with a splitting ratio of 50/50. In the same year, 500 m PMF and PMF optical ring mirrors were utilized to realize a linearly polarized RFL, in which the polarization extinction ratio of output is up to 25 dB [93]. In 2017, a Brillouin RFL based on PMF obtained a high-efficiency output, of which the polarization extinction ratio is higher than 25 dB [94,95]. Thus, the polarization extinction ratio and the conversion of optical quantum efficiency of the output emission can be enhanced by applying PMF into the RFL system.

Moreover, these mentioned optical fibers, research on other fiber-based RFLs is investigated widely. For example, the RFL formed by MMF [96] could cut the spectrum of output [97], the RFL based on TOF can emit high power lasing [51], and TWF-based RFL can generate controllable double wavelength random lasing [98]. It means that we can fulfill different applications according to different RFLs formed by fitting fiber.

## 3. Control of Output Characteristics

Having experienced vigorous development, RFLs mainly concentrate on a few hot aspects: Output power, wavelength, and line-width. Many researchers have made contributions to optimize the output performance of RFLs.

### 3.1. Output Power

High power RFLs are an important orientation in optical fields. The power of the RFL is only a few watts at first [47], but it becomes in kilowatts level after experiencing rapid progress in nearly a decade [99].

**Figure 9:**
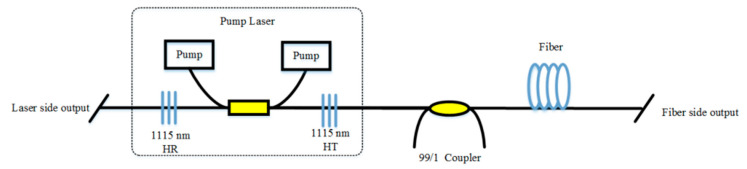
The scheme of RFL pumped by ytterbium-doped fiber laser (YDFL). HR, high reflection; HT, high transmission.

**Figure 10:**
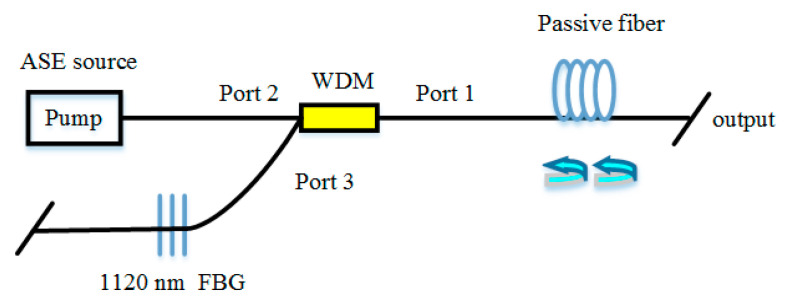
The scheme of RFL pumped by an amplified spontaneous emission (ASE) source.

At first, the pump source that has high power and high-efficiency of light conversion is adopted to improve the output power of RFLs. Ytterbium-doped fiber laser (YDFL) is a decent choice for the pump source [100]. In 2011, 7.5 W YDFL was used to pump 10.7 km TWF for the first time, and only 3.8 W emission was obtained, the scheme of RFL pumped by YDFL is shown in Figure 9 [47]. By 2016, a single-terminal RFL of 112 W output with 84.8% light conversion efficiency was observed, which used YDFL to pump a half-open-cavity RFL comprised of 320 m long passive fiber and an FBG [64]. Furthermore, using an incoherent pump to obtain high power RFL was proposed. In 2017, a hundred-watt linear polarization RFL was generated by using a broadband ASE source as the pump, and the second-order Stokes light that centered at the wavelength of 1178 nm reached the maximum power of 100.7 W. Moreover, the theoretical result suggested that the RFL with an incoherent pump could get 300 W linear polarization single-mode first-order Stokes light [67]. In 2019, the maximum power of the first-order Stokes light was up to 117 W, by optimizing the bandwidth of incoherent ASE pump source (the scheme of RFL is pumped by ASE source is depicted in Figure 10) [68]. However, the problems are the power of the incoherent pump is usually low, and amplifiers that result in the complexity of the RFL structure are required.

**Figure 11:**
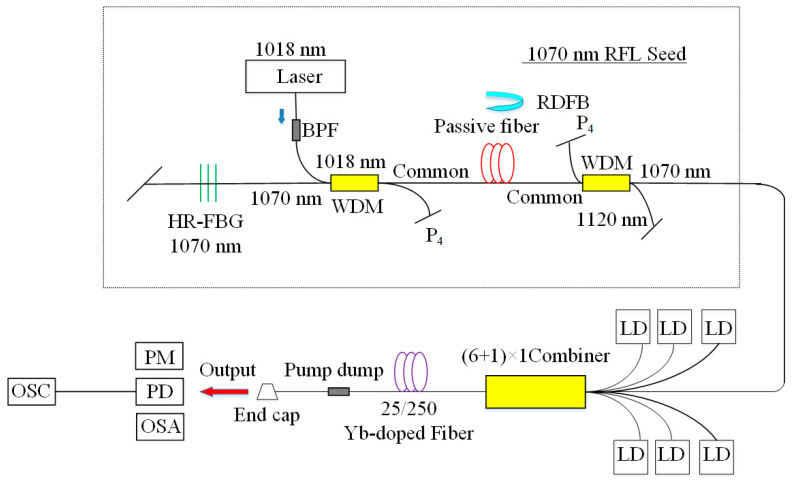
The setup of the RFL seed and the amplifier. BPF, bandpass filter; P4, an extra port of WDM; RDFB, random distributed feedback; HR-FBG, high-reflectivity fiber Bragg grating; LD, 976 nm laser diode; PD, photo-detector; OSC, oscilloscope.

An alternative approach to improve the output power is by optimizing the gain. Although Raman gain [101] is a considerable principle to realize high power RFL rather than the Brillouin gain, the main oscillator power amplifier (MOPA) [102], which has a much higher gain, is a typical way. The traditional MOPA system has a nonlinear spectral broadening phenomenon, and the line-width of output remains stable throughout the high power amplification process. However, it is of profound significance for further power scaling in the spectral domain to suppress the broadening of line-width, as the combination of spectral beams requires narrow line-width and high power laser. In 2015, a high power RFL with kilowatt-level was observed, which utilized the MOPA structure (Figure 11 shows the setup of the RFL seed and the amplifier.) [103]. In 2017, a high power linearly polarized RFL based on the structure of the MOPA was reported. Near-diffraction limited beam quality and high polarization of 93.9% were obtained under the maximum output power [104]. In 2019, random lasing emission with the highest power of 3.03 kW was achieved by using the structure of tandem-pumped MOPA [105]. Using the same medium of gain, Wang, Z. et al. presented a monolithic high power RFL with a tandem-pump scheme and obtained 4.4 kW output power [99]. This is the ever report of the highest power RFL. It is worth noting that although the power of the RFL reaches up to kilowatts level in this approach, the structure is much more complicated. Table 1 summarizes some of the evolution of high power RFL utilizing these approaches.

### 3.2. Wavelength

Research on the wavelength of the RFL involves tunability and multi-wavelength, as the wavelength of the RFL can design flexibly.

**Figure 12:**
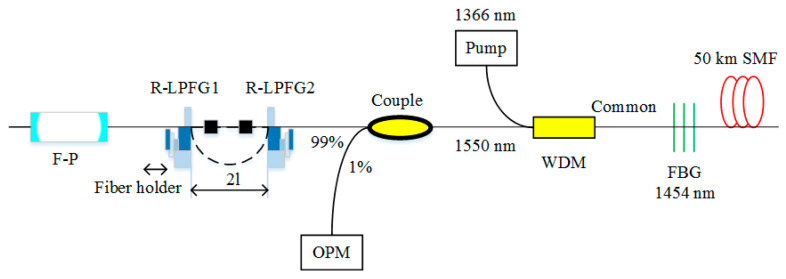
Experimental setup for the tunable multi-wavelength RFL.

Tunable modules are fundamental structures to achieve tunable RFLs. The continuous tunable random laser was realized from 1553.9 nm to 1565.4 nm in 2013 [106], by combining SMF and multi-channel wavelength-tunable elements: F-P cavity and Mach-Zehnder interferometer formed by long-period gratings. The experimental setup for the tunable multi-wavelength RFL is illustrated in Figure 12. In 2014, a tunable erbium-doped RFL based on the random distributed feedback of backward Rayleigh scattering went up to 40 nm (1525~1565 nm) of the range of tunable wavelength, by connecting 20-km-long SMF and tunable fiber F-P interferometer filter [83]. In 2017, broadband tunable single-mode RFL with a range of more than 1500~1570 nm was realized, having adopted a tunable F-P filter [107]. These performances open the further prospect for applications in sensing and optical communication.

**Figure 13:**
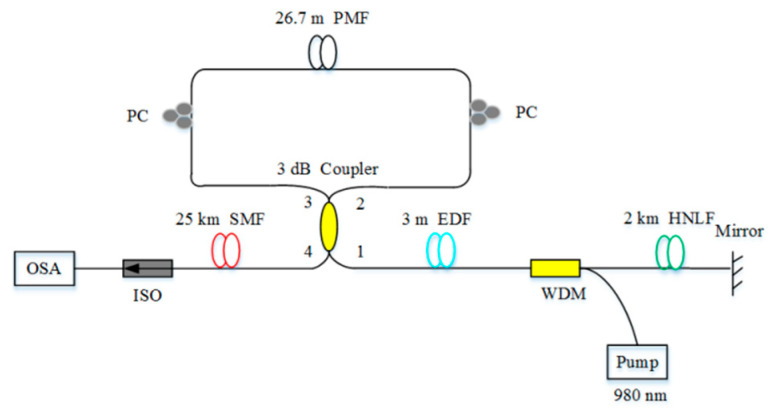
Experimental setup of multi-wavelength erbium-doped RFL. ISO, isolator; HNLF, high nonlinear optical fiber.

**Figure 14:**
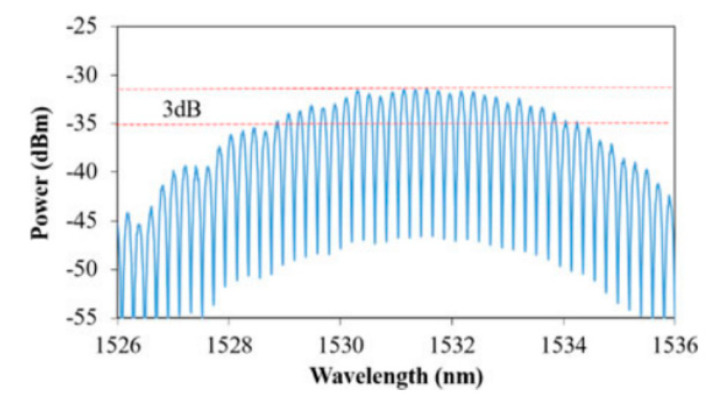
Generation of 24 channels within 3-dB flatness [109].

Also, multi-wavelength lasers have potential applications in optical fiber sensing. RFLs with multi-wavelength can be realized by adding an array of FBGs, Sagnac loop filter, Lyot filter, as well as cascaded Brillouin conversion. In 2017, Bao et al. proposed a narrow-line-width dual-wavelength RFL based on semiconductor optical amplifier (SOA) and FBGs [108]. In 2018, an erbium-doped RFL was achieved with stable multi-wavelength. The experimental setup of multi-wavelength erbium-doped RFL is depicted in Figure 13. Moreover, the experimental results showed that 24 stable laser lines can be generated in the range of 3 dB flatness when the pump power is 350 mW (Figure 14) [109]. In 2020, multi-wavelength (19 laser lines) laser output with flat amplitude was obtained by using a tunable incoherent optical pump and Lyot filter [110].

### 3.3. Line-Width

**Figure 15:**
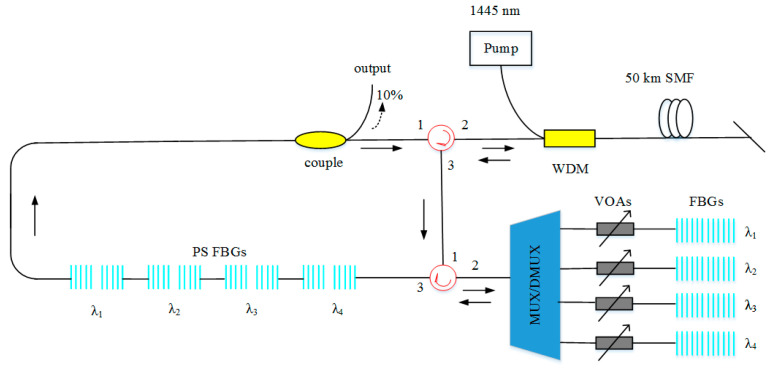
Setup of the multi-wavelength RFL. PS FBGs, phase-shifted fiber Bragg gratings; VOA, variable optical attenuators; MUX/DMUX, multiplexer/demultiplexer.

Narrow line-width RFL performs excellent coherence properties, and is widely used in ultra-high-precision laser radar, inter-satellite communication, as well as spacecraft docking. Utilizing filer involving fiber F-P (FFP) or FBG could obtain an RFL with 0.05 nm line-width [111]. Moreover, combining phase-shifted FBGs and regular FBGs is a feasible method to obtain spectral narrow line-width RFL (Figure 15 shows the setup of the multi-wavelength RFL.) [29]. Although these setups could be constructed easily and narrow down the line-width of the RFL, the coherence cannot be improved, due to the intrinsic factor of the RFL, and the line-width is limited to narrow down further.

**Figure 16:**
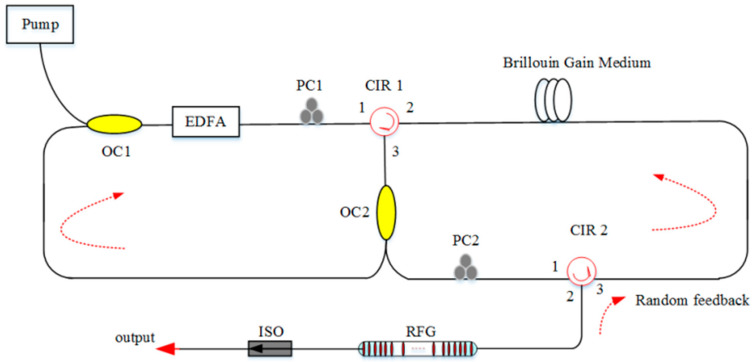
Experimental setup of RFL. OC, optical coupler; CIR, circle.

The gain mechanism is an intrinsic factor for narrowing the line-width, which is mentioned in Section 2.2. Moreover, random feedback is an inner factor that can influence the line-width of the RFL. In 2014, 2.1 kHz narrow line-width RFL was proposed, which was based on random gratings that can strengthen random feedback [112]. In 2016, an RFL with the line-width of 1.17 kHz was realized by using Rayleigh-enhanced TOF [113]. In 2017, an RFL with the line-width of 1 kHz was presented, in which a 5 km non-uniform fiber provided an enhanced feedback strength for the random lasing resonance [108]. In 2018, enhanced random feedback from RFGs along the 25 km long-span half-open-cavity RFL was proposed (Figure 16), obtaining more Stokes channels with sub-kHz line-width [114].

## 4. Sensing Application

The optical fiber [115] is widely used in geophysical fields, such as oil and gas exploration, tunnel safety monitoring, and low-frequency seismic monitoring because of low cost and insensitivity to electromagnetic interference. It can get into places inaccessible to people, such as high-temperature regions, or places harmful to human health. RFLs that have long-distance optical fibers can be applied in the sensing system, as they have high sensitivity. This section introduces two aspects of optical fiber sensing of the RFL: Point-sensing and distributed-sensing, which are based on the role played by optical fiber in the sensing system.

### 4.1. Point-Sensing

The sensing element requires over 100 km away from demodulation devices [21,116] to apply ultra-long-distance safety detection (electric-lines, oil, and gas pipelines). FBG, a passive device, which is applied to the composition of RFL, could satisfy the practical requirements. As the external environment changes, the spectrum of FBG changes, and the spectrum of RFL will change accordingly. Because the fiber in the sensing system only plays the role of light transmission, the information to be measured is determined by the central wavelength of the FBG sensor.

For example, a Raman RFL sensor for strain-temperature measurement was presented in 2009 [117]. FBGs remotely located at a distance of 10 km were characterized by the strain and temperature. The proposed system could measure strain and temperature simultaneously, using FBGs with low reflectively. The resolutions of temperature and strain are ±0.1 °C and ±12.6 *με*, respectively.

**Figure 17:**
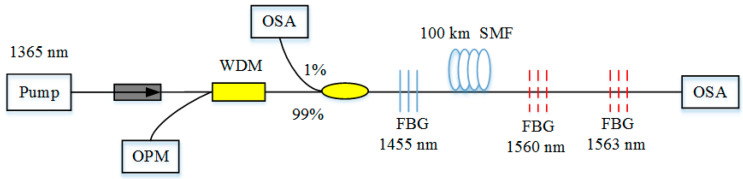
Scheme of RFL using FBG as a sensing element.

However, the sensing distance is only 10 km, which is far from satisfying the demand. Long-distance optical fiber remote sensing (beyond 100 km) was achieved in 2012, which is a mandatory requirement for sensing in harsh environments. This system was reported by Wang, which utilized first-order random lasing and second-order random lasing, as shown in Figure 17. For the first-order scheme, RFL is pumped by a 1365 nm pump source, while an FBG sensor whose central wavelength is 1455 nm corresponding to the first-order Stokes light is placed at the near end of the fiber span. For the second-order scheme, generated first-order Stokes light is the source of pumping. An FBG sensor with the central wavelength of 1560 nm, which is corresponding to the second-order Stokes light, is placed at the far end of the fiber span. The wavelength of output would shift according to the change of temperature of the FBG—namely, the retrieval of the sensing parameter only depends on the central wavelength of the FBG sensor, which makes the system robustness against degradation of the pump source or fiber span [43]. Moreover, for the first-order random lasing and second-order random lasing, the SNR is as high as 20 dB and 35 dB, respectively, which is sufficient for sensing at a distance of 100 km. Thus, this scheme is attractive for practical applications. However, the pump power of RFL is relatively high (>1.7 W), due to the Raman gain. After that, they optimized the scheme of long-distance remote sensing [118], having placed the AOF in the middle of two SMF segments and taken the reverse RFL pumping scheme. The sensing distance is longer than that of the setup without AOF, and the SNR is over 30 dB. With a similar method, a point sensing RFL system of an ultra-long-distance of 200 km was obtained in 2013 [119]. This scheme can multiplex 11 FBGs, and the SNR is high enough to observe output emission. Thus, it has the capability of the multiplex.

In 2016, a high-resolution point sensing RFL system with better stability was attained [45], in which the temperature sensitivity is less than 0.01 °C, and the strain sensitivity is less than 0.2 *με*, and the resolution could be further increased by combining this system and improving-FBGs. To further decrease the threshold of the pump, Jaharudin et al. introduced a remote temperature sensor by adopting a single-wavelength erbium-doped RFL [120]. In 2019, a high-resolution static strain RFL sensor was proposed with RFGs, and a high precision π-phased shifted FBG. The static strain resolution is 196 *pε* in a period of 160 s [121]. However, the scheme is incredibly complex, and the operation is difficult.

**Figure 18:**
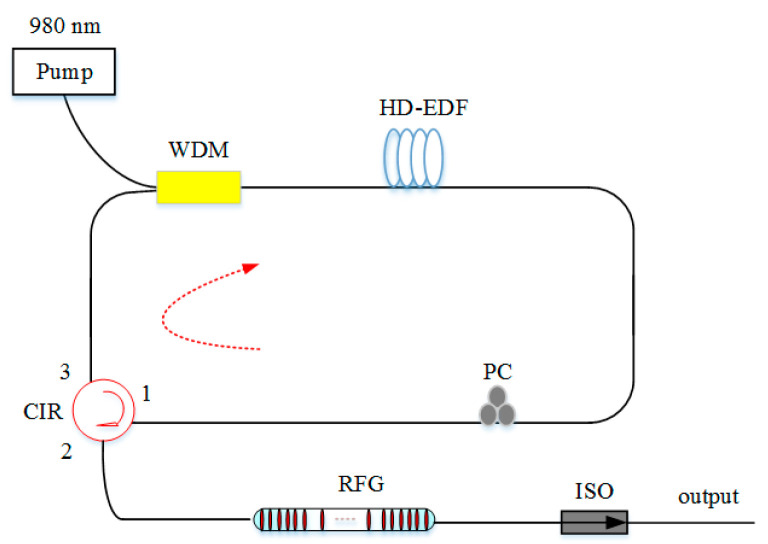
Setup of RFL based on the strong RFG for ultrasound detection.

Moreover, ultrasound sensing is a valuable technique, which can be widely used in the areas of ultrasound-related, such as biomedicine, geological disaster surveillance, material testing, dimensional measurement, as well as structural health monitoring. This is partly because the detection of inaccessible parts and the real-time structural monitoring are impossible for conventional methods. Regular FBGs have been extensively studied for ultrasound sensing [122,123], but the broadband reflection of FBGs suffers week response from broad bandwidth light source in the ultrasound sensing. The RFL based on RFGs is a better choice for ultrasound detection. As RFGs can offer strong random scattering and broadband spectral reflection. In 2017, RFGs were used as the ultrasound sensing head for the first time; meanwhile, they provided random feedback for the RFL [124]. As long as the effective length of the grating can be less than the wavelength of the ultrasound waves, ultrasound sensing would be realized. It proved that this RFL based on RFGs realized broadband ultrasound detection of 0.8 MHz with high SNR and responded to the harsh environment with variations of large temperature and strain. To improve the sensitivity, reduce the cost, and optimize the structure, a high-efficiency RFL based on RFGs was proposed in 2020 [125], making it possible for the high response of temperature and strain, as well as broad ultrasound bandwidth of 8 MHz. The setup of RFL based on the strong RFG for ultrasound detection is depicted in Figure 18. This configuration is implemented by a lasing resonance with high doped EDF (HD-EDF), which is pumped by a 980 nm laser through a WDM. The HD-EDF can effectively reduce the threshold of the pump source and the length of fiber with strong gain because of the high erbium concentrate. The RFG provides random feedback, and could be as a sensing head. It greatly broadens the bandwidth of sensing and shows the practical applications in ultrasound sensing.

In the point-sensing system, gratings are the important passive devices, due to the high sensitivity for the change of the environment. They play the role of sensing elements, as well as provide feedback for RFLs. Thus, RFLs based on the FBGs and RFGs can apply in the remote sensing system, and ultrasound sensing system, respectively. FBGs and RFGs would perform broad bandwidth, high stability, high sensitivity, flexible multiplexing capability, and robustness in the sensing system. However, gratings always require the etching technique artificially controlled. There is no doubt that the process of forming an RFL system has increased in complexity and difficulty.

### 4.2. Distributed-Sensing

RFLs based on long-distance fiber are perfect mediums for distributed sensing application, which can be used to improve the SNR in the distributed sensing system, because they are insensitive to the changed information of physical parameters, such as temperature and strain along the fiber. Currently, it is mainly used in the Brillouin optical time-domain analysis (BOTDA) system.

**Figure 19:**
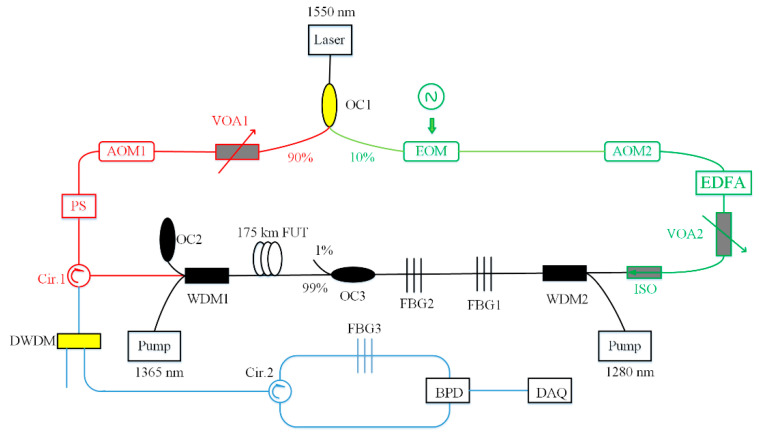
Experimental setup of 175 km BOTDA. EOM, electronic-optical modulator; AOM, acoustic optical modulator; FUT, fiber under test; BPD, balanced photo-detector; DAQ, data acquisition.

**Figure 20:**
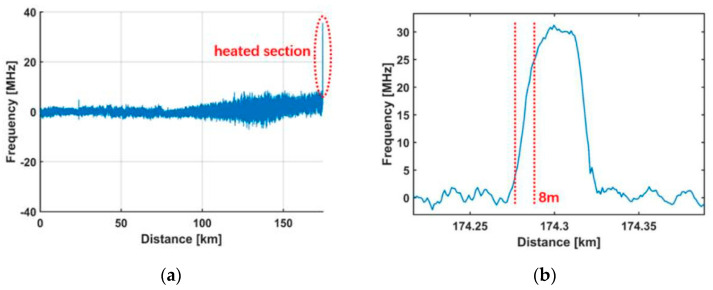
BFS difference versus distance. (**a**) Along the whole fiber; (**b**) around the heated section [127].

An ultra-long distance distributed BOTDA sensing system was demonstrated in 2013 [126]. The experiment adopted a mixed composition of a second-order pump based on the RFL and a first-order pump based on the low-noise laser diode. The hybrid pump could optimize the gain distribution and extend the transmission distance. The results showed the repeaterless sensing distance is 154.4 km with 5 m spatial resolution and ±1.4 °C measurement uncertainty. In this system, the pulses and probe light need to maintain certain SNR, and high SNR is beneficial to the extension of the sensing distance. There is no doubt that higher pump power is required for second-order emission, but it would result in the instability of modulation and obvious nonlinear effect. Relative intensity noise would cause the impairment of SNR. Thus, it is still a challenge for RFLs in the distributed sensing system. However, in 2019, due to optimizing the pump source with the second-order and third-order Raman laser, the sensing distance was further increased [127]. The experimental setup of 175 km BOTDA is shown in Figure 19. There are four configurations in this setup. The red part indicates the production of the pulses in BOTDA, and the green part presents the generation process of the probe light. The black part is the FUT that is also a part of RFL, and the blue part shows the detection module. In detail, the second-order Raman laser (1365 nm) is at the left of the FUT, and the third-order Raman laser (1280 nm) is at the right of the FUT. Both of them are pump sources. FBG1 and FBG2 are centered at the wavelength of 1461 nm and 1365 nm, and their bandwidths are 0.627 nm and 0.586 nm, respectively. The reflectivity of both is above 90%. This scheme realized a 175 km repeaterless BOTDA system with 8 m spatial resolution and ±2.06 MHz uncertainty (Figure 20). This has been the longest distance of repeaterless BOTDA reported up to date.

These schemes are all based on the hybrid pump, which have higher SNR compared with systems that adopted forward pump source. This is because the RFL possesses a lower effective noise figure in these configurations. Moreover, high-order RFL would perform stable gain distribution, avoiding power fluctuation along the transmission fiber caused by EDFA or distributed Raman amplification in the distributed sensing system. What’s more, RFL is insensitive to the variation of temperature, making the BOTDA sensing system much more stable.

As the distance of fiber sensing increases with the existence of the RFLs, RFLs could be applied to the safety monitoring of important facilities related to the national economy and people’s livelihood: Bridges, tunnels, oil, and gas pipelines, as well as expressways. Moreover, the high SNR and the less gain fluctuation can be obtained, due to the RFLs, which is of vital importance in the sensing system. However, there are a few studies on point-sensing and distributed-sensing, which are based on RFLs. Challenges of huge loss caused by the ultra-long optical fiber and spectral broadening caused by the nonlinear effect would be required to overcome.

## 5. Conclusions

In the last decade, researchers have proposed different structures of RFLs. RFLs based on the AOF or the special optical fiber are gradually explored, not only commercial SMF. This means gain mechanisms are not only including passive gain, but also including active gain and hybrid gain. Moreover, more properties of the RFL have attracted much attention from researchers, such as polarization, dispersion, and nonlinear effects.

Through reviewing the progress of the RFL’s output emission in the past ten years, it is found that hot areas of the RFLs involve output power, wavelength, and line-width. Approaches of improving output characteristics have been developed greatly, including the aspect of the pump, gain, and feedback, which would drive the further development of core technology in related areas.

The practical application is an important orientation for the RFL, such as optical fiber sensing. In the RFL system, long-distance sensing can be used to achieve the purpose of safety monitoring, regardless of whether the optical fiber plays the role of light transmission or sensing.

However, the RFL still requires further theoretical and experimental exploration. It could be worth exploring other pump sources, except for ASE or narrow line-width lasers, as they could be utilized to optimize the output characteristics of the RFL. The random feedback of most RFLs is provided by Rayleigh scattering, which is quite weak in the scheme, and a few investigations on improving the strength of random feedback have been finished. The physical properties of the output beam have yet to be completed. Furthermore, the potential application of RFLs could be explored in optical fiber sensing.

## Figures and Tables

**Table 1:** The evolution of high power RFL.

Year	Approach	Pump Power	Output Power
2011	YDFL	7.5 W	3.8 W
2014	YDFL	10 W	7 W
2014	YDFL	98.6 W	73.7 W
2015	YDFL	221.4 W	193.5 W
2015	MOPA	1381.4 W	1.03 kW
2017	ASE source	127 W	104.8 W (second-order)
2019	MOPA	3.61 kW	3.03 kW
2019	YDFL	1248 W	985 W
2019	MOPA	4.343 kW	4.02 kW
